# A Tumor‐Boundary Niche Enriched for PD‐1^+^CD8^+^T Cells and BTLA^+^B Cells Drives Resistance to Immunotherapy in NSCLC

**DOI:** 10.1002/advs.77812

**Published:** 2026-09-27

**Authors:** Jiajuan Wu, Shiying Guo, Leilei Lv, Guiqin Fan, Yu Shen, Qiuxia Qu, Cheng Chen

**Affiliations:** ^1^ Department of Pulmonary and Critical Care Medicine The First Affiliated Hospital of Soochow University Suzhou China; ^2^ Clinical Immunology Institute The First Affiliated Hospital of Soochow University Suzhou China; ^3^ Department of Clinical Laboratory The Affiliated Suzhou Hospital of Nanjing Medical University Suzhou Municipal Hospital Gusu School Nanjing Medical University Suzhou China; ^4^ Department of Pulmonary and Critical Care Medicine The Fourth Affiliated Hospital of Soochow University Suzhou China; ^5^ Department of Pulmonary and Critical Care Medicine The Taicang Hospital of Nanjing University, Taicang Traditional Chinese Medicine Hospital Suzhou China

**Keywords:** B cells, BTLA, CD8^+^T cells, immunotherapy, NSCLC, PD‐1, TME

## Abstract

CD8^+^T cells are central mediators of antitumor immunity, and their functional heterogeneity profoundly influences immunotherapy efficacy. We characterized the spatial distribution of CD8^+^T cell subsets stratified by PD‐1 expression within the tumor microenvironment (TME) of non‐small cell lung cancer (NSCLC). Our analyses revealed that PD‐1^+^CD8^+^T cells were more prevalent in the non‐central regions than in the tumor core, and higher abundance of these cells in the non‐central zone was associated with poorer response to PD‐1 blockade. Enrichment of PD‐1^+^CD8^+^T cells diminished cancer cell apoptosis induced by anti‐PD‐1 mAb treatment in an in vitro co‐culture system modeling in vivo conditions. The PD‐1^+^CD8^+^T cell subset exhibited significantly reduced clonal diversity, as determined by TCR Vβ repertoire analysis, single‐cell T cell receptor sequencing (scTCR‐seq), and GLIPH2 clustering, along with impaired effector functions and distinct transcriptional programming. Mechanistically, IL‐6 produced dominantly by non‐central tumoral BTLA^+^B cells promoted PD‐1 expression on CD8^+^T cells, mirroring the spatial pattern in tumoral PD‐1^+^CD8^+^T cells. Our data further identify a spatially organized functional unit comprising PD‐1^+^CD8^+^T cells and neighboring BTLA^+^B cells within the non‐central tumor region. This specialized niche, defined by anatomical localization, functional state, TCR repertoire, and intercellular crosstalk, contributes to the establishment of resistance to immune checkpoint blockade.

## Graphical abstract

A spatial niche composed of BTLA^+^B cells and CD8^+^T cells is enriched at the tumor boundary in non‐small cell lung cancer. Within this specialized niche, BTLA^+^B cells act as a dominant source of IL‐6, upregulating PD‐1 on CD8^+^T cells via STAT3 signaling. This spatially organized immune unit enhances understanding of heterogeneous immune cell interactions and contributes to resistance to checkpoint blockade.

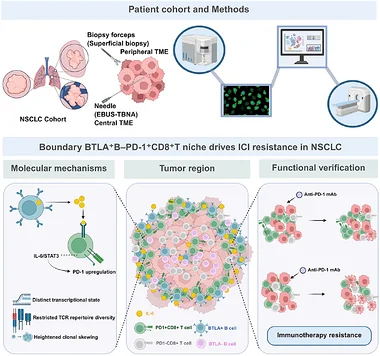

## 1 Background

NSCLC is one of the leading causes of cancer‐related mortality worldwide [1]. In recent years, immunotherapy has been regarded as a very promising therapeutic strategy for NSCLC, focusing on reactivating the immunosuppressive environment induced by cancer cells and improving antitumor immune responses [2, 3]. Notwithstanding these developments, there are still a number of obstacles to the therapeutic use of immune checkpoint inhibitors (ICIs) [4], such as low response rates and possible immune‐related side effects, which call for more research to maximize therapy results.

Numerous immunosuppressive characteristics of the TME have a substantial impact on the effectiveness of immunotherapy [5, 6]. Among them, the effectiveness and longevity of anticancer immune responses are significantly influenced by the functional subset and spatial infiltration of CD8^+^T cells, which are the primary effector population in cell‐mediated antitumor immunity within this immunologically complicated environment [7, 8, 9]. The infiltration of CD8^+^T cells and cellular interaction are critical for PD‐1 blockade therapy [10, 11].

Besides, chronic inflammatory cytokine stimulation and prolonged exposure to tumor antigens often cause CD8^+^T cells to gradually lose their ability to fight tumors, which ultimately compromises their ability to do so [12, 13, 14]. Specifically, existing studies that examine the prognostic impact of CD8^+^T cell infiltration in GC are inconsistent, indicating the heterogeneity of intratumoral CD8^+^T cells [15, 16]. Notably, the functional distribution and activation status of invading CD8^+^T cells, especially PD‐1 expression patterns among these immunological populations, should be given special consideration in light of these pathways.

To better explore the spatial organization of the PD‐1^+^CD8^+^T cell subset clustering niche and uncover intrinsic mechanisms of resistance to ICIs, we developed analytical biopsy techniques to systematically characterize the tumor microenvironment (TME) in non‐small cell lung cancer (NSCLC). We discovered that PD‐1^+^CD8^+^T cells were selectively co‐localized and accumulated with BTLA^+^B cells in the non‐central regions of the tumor. This spatial niche provides insight into potential strategies for patient stratification in personalized therapy and suggests a possible association between immunotherapy resistance and region‐specific immune profiles. Consistent with these findings, analysis of single‐cell T cell receptor sequencing (scTCR‐seq) further revealed that reduced TCR repertoire diversity was largely confined to the PD‐1^+^CD8^+^T cell subset, thereby impairing the breadth and potency of antitumor immunity induced by anti‐PD‐1 mAb.

## 2 Methods

### 2.1 Patients

A cohort of 105 patients diagnosed with lung cancer was enrolled at the First Affiliated Hospital of Soochow University between 2022 and 2024. None of the participants had received any form of anti‐tumor therapy prior to sample collection. The patient cohort comprised 87 males (82.86%) and 18 females (17.14%), with a mean age of 67.38 years (range: 37–87 years). Comprehensive clinical information, including age, sex, tumor size, histological subtype, treatment regimen, and therapeutic outcome, was retrospectively collected for all participants. Treatment allocation was not randomized but was determined according to standard clinical protocols and treatment availability at the institution. This study was approved by the Institutional Review Boards of The First Affiliated Hospital of Soochow University (2019‐070 and 2024‐465), and written informed consent was obtained from all participants prior to sample collection.

### 2.2 Acquisition of Tumor Samples

Tumor tissue specimens were obtained via bronchoscopy. When the neoplasm was directly visible during bronchoscopy, a superficial forceps biopsy was performed under endoscopic guidance. For suspected lung cancer lesions that were not visible on conventional bronchoscopy, intratumoral endobronchial ultrasound‐guided transbronchial needle aspiration (EBUS‐TBNA) was performed using a 22‐gauge needle. All enrolled patients completed the specimen collection phase with no loss to follow‐up.

### 2.3 Isolation of Mononuclear Cells From Lung Cancer Tissue

Fresh tissue specimens were dissected and immediately immersed in RPMI 1640 medium (Gibco, USA). Tissues were initially mechanically dissociated using sterile scissors, followed by enzymatic digestion at 37°C for 30 min with a solution containing DNase I (0.3 mg mL^−1^; Sigma–Aldrich) and Liberase TL (0.2 mg mL^−1^; Roche) in serum‐free RPMI 1640 medium. After digestion, the cell suspension was passed through a 70‐µm cell strainer. For specimens obtained via intratumoral EBUS‐TBNA, red blood cell lysis was performed by incubating cells with 10 mL ACK lysis buffer (Beyotime Biotechnology, China) at room temperature for 10 min. Finally, the isolated cells were resuspended in 1 mL of flow cytometry staining buffer and prepared for flow cytometric analysis.

### 2.4 Flow Cytometry (FCM)

Single‐cell suspensions were prepared at a concentration of 2 × 10^6^ cells mL^−1^ before antibody staining. The cells were stained with a panel of fluorescently‐conjugated mAbs for 30 min at 4°C in the dark. The antibody panel included APC/Cyanine7‐conjugated anti‐human CD45 (clone HI30, Cat# 304014), PE/Cyanine7‐conjugated anti‐human CD8α (clone SK1, Cat# 344750), FITC‐conjugated anti‐human CD20 (clone S18015E, Cat# 375508), PE‐conjugated anti‐human BTLA (clone MIH26, Cat# 344506), and APC‐conjugated anti‐human PD‐1 (clone EH12.2H7, Cat# 329907), all purchased from BioLegend. Fluorescence minus one (FMO) controls were utilized to establish precise gating boundaries. Lymphocytes were initially gated based on forward and side scatter properties (FSC‐A vs SSC‐A). Subsets of CD20^+^BTLA^+^B cells and PD‐1^+^CD8^+^T cells were further gated within the CD45^+^ leukocyte population. Comprehensive gating strategies are depicted in Figure 5D and Figure S4A.

For intracellular cytokine staining, freshly isolated single‐cell suspensions were subjected to ex vivo stimulation in complete RPMI 1640 medium containing 50 ng mL^−1^ phorbol 12‐myristate 13‐acetate (PMA, Sigma–Aldrich) and 500 ng mL^−1^ ionomycin (Sigma–Aldrich) at 37°C for 1 h. Brefeldin A (1 µg mL^−1^, Sigma–Aldrich) was subsequently added for an additional 3 h to block intracellular cytokine secretion. Following stimulation, cells were fixed and permeabilized using the Intracellular Fixation & Permeabilization Buffer Set (Thermo Fisher Scientific). Cells were then intracellularly stained with PE/Cyanine7‑conjugated anti‑human IL‑6 mAb (clone MQ2‑13A5, BioLegend, Cat# 501119) to assess IL‑6 production. Flow cytometry was performed on a Beckman Coulter Navios flow cytometer, and data were analyzed with FlowJo software (Tree Star).

### 2.5 Analysis of the TCR‐Vβ Repertoire Usage

TCR‐Vβ repertoire usage in PD‐1^+^ and PD‐1^−^CD8^+^T cell subsets were assessed by flow cytometry employing the IOTest BetaMark TCR Repertoire Kit (Beckman Coulter, Marseille, France). This kit contains a panel of monoclonal antibodies enabling the simultaneous detection of 24 distinct TCR‐Vβ subfamilies. Each TCR‐Vβ subfamily is targeted by a cocktail of three monoclonal antibodies conjugated to FITC, PE, or a combination of both fluorochromes. The mean usage frequency and standard deviation (SD) for each TCR‐Vβ subfamily were calculated to compare repertoire differences between PD‐1^+^ and PD‐1^−^CD8^+^T cells. Raw TCR‐Vβ usage values were subsequently Z‐score normalized and expressed in SD units. Comparative heatmaps were generated to visualize the TCR‐Vβ repertoire landscape, with a color gradient from green to red denoting values ranging from ‐1 SD to > +2 SD relative to the mean expression level for each TCR‐Vβ family.

### 2.6 Multiplex Immunofluorescence Analysis

Surgically resected NSCLC tissue sections were deparaffinized, rehydrated, and subjected to heat‐induced antigen retrieval in citrate buffer. After blocking with 3% bovine serum albumin (BSA) at 37°C for 1 h, the sections were incubated overnight at 4°C with primary antibodies, including anti‐human PD‐1 mAb (1:100, ZSGB‐Bio, Cat# TA806807), anti‐human CD8 mAb (1:300, ZSGB‐Bio, Cat# ZA‐0508), anti‐human BTLA mAb (1:500, Abcam, Cat# ab181406), anti‐human CD20 mAb (1:100, Abcam, Cat# ab78237), anti‐human IL‐6 mAb (1:400, Abcam, Cat# ab6672), anti‐human CD68 mAb (1:300, Abcam, Cat# ab213363), and anti‐human FAP mAb (1:200, Cell Signaling Technology, Cat# 52818).

Multiplex staining was performed sequentially with tyramide signal amplification (TSA). In each staining cycle, the primary antibody was detected with a horseradish peroxidase (HRP)‐conjugated secondary antibody, and HRP catalyzed the deposition of a fluorophore‐conjugated tyramide that covalently bound to tyrosine residues in the vicinity of the target antigen. The primary‐secondary antibody complex was then stripped by microwave heating in antigen retrieval buffer, while the covalently deposited fluorescent tyramide signal remained intact. This cycle was repeated for each subsequent marker using spectrally distinct fluorophores. After all targets were sequentially labeled, nuclei were counterstained with 4′,6‐diamidino‐2‐phenylindole (DAPI). Immunofluorescence images were acquired using matched spectral filters on the Akoya Mantra 2 Quantitative Pathology Workstation. To quantify target cell abundance, three representative non‐overlapping high‐power fields of view (200× magnification) were selected per case. Target‐cell numbers were counted in each field of view using QuPath 0.7.0, and the mean value was determined for each case from these three fields.

### 2.7 Proximity Analysis of Tumoral BTLA^+^B Cells to PD‐1^+^CD8^+^T Cells

The HALO platform was employed to calculate the nearest‐neighbor distances between distinct immunophenotyped cell subsets. In detail, the proximity of the BTLA^+^ or BTLA^−^B cells to the PD‐1^+^ or PD‐1^−^CD8^+^T cells was analyzed using the HALO Spatial Analysis Module. Within this module, cells were classified according to immunophenotype, and a spatial distribution plot was generated for each sample. The proximity analysis tool was applied to evaluate cellular interactions occurring within a 100 µm distance threshold.

### 2.8 In Vitro Culture Assay

Human lung cancer tissue specimens were collected in RPMI 1640 medium with 5% FBS and 1% penicillin‐streptomycin and transported on ice. After removal of necrotic tissue and washing with PBS, tissues were mechanically minced and digested in serum‐free RPMI 1640 medium containing DNase I and Liberase TL. The digested mixture was filtered and centrifuged to obtain a single‐cell suspension. Isolated cells were divided into two experimental groups treated with complete medium alone (untreated control) or IL‐6 (20 ng mL^−1^, MCE, Cat# HY‐P7044). For additional inhibition experiments, the STAT3 inhibitor Stattic (5 µM, MCE, Cat# HY‐13818) was supplemented to the culture system. Following 72 h of incubation, cells were assessed by FCM after staining with anti‐human CD45, anti‐human CD8, and anti‐human PD‐1 mAbs, as described in Section 2.4.

### 2.9 Assay for Apoptosis

Fresh lung cancer cells were suspended in complete medium and adjusted to a concentration of 8 × 10^5^ cells mL^−1^. A 50 µL aliquot of the cell suspension was seeded into each well of a 6‐well plate, followed by the addition of 2 mL of complete medium. The cells were then randomly assigned to two experimental groups: (i) treatment with human anti‐PD‐1 mAb (10 µg mL^−1^, Selleck, Cat# A2005) and (ii) complete medium alone (untreated control). After a 72 h incubation at 37°C under 5% CO_2_, the cells were stained with APC‐conjugated anti‐human CD45 mAb (clone HI30, BioLegend, Cat# 304012) and BV421‐conjugated anti‐human EpCAM mAb (clone 9C4, BioLegend, Cat# 324219) for 20 min at 4°C in the dark. Following staining, the cells were centrifuged, the supernatant was removed, and the cell pellet was resuspended in 100 µL of 1× Binding Buffer. To assess apoptosis, 2.5 µL of FITC‐conjugated Annexin V and 2.5 µL of propidium iodide (PI) were added to each sample, which was then incubated in the dark at room temperature (25°C) for 15 min. Subsequently, 200 µL of 1× Binding Buffer was added to each sample, and flow cytometric analysis was performed within 1 h.

### 2.10 Assessment of Immunotherapy

A cohort of 43 patients diagnosed with NSCLC received ICIs as first‐line therapy. Antitumor efficacy was evaluated by reviewing computed tomography (CT) scans obtained after two treatment cycles. Tumor response was independently assessed by two radiologists who were blinded to clinical information to minimize bias. Patients achieving complete response (CR) or partial response (PR) were classified as responders (objective response rate, ORR), while those with stable disease (SD) or progressive disease (PD) were categorized as non‐responders. Changes from baseline in the sum of the longest diameters of target lesions were assessed according to the Response Evaluation Criteria in Solid Tumors (RECIST) version 1.1 guidelines.

### 2.11 Single‐Cell RNA Sequencing (scRNA‐seq) and scTCR‐seq Library Preparation

Single‐cell suspensions were processed on the 10x Genomics Chromium Controller to generate gel beads‐in‐emulsion (GEMs). Cells were co‐encapsulated with barcoded gel beads and reverse‐transcription reagents. Following on‐bead cDNA synthesis, emulsions were broken; barcoded full‐length cDNA was amplified and purified. For each sample, two libraries were constructed: a V(D)J‐enriched TCR library and a 5’ gene‐expression library. All steps followed the manufacturer's recommendations.

### 2.12 Processing of scRNA‐seq and scTCR‐seq Data

Raw FASTQ files were processed with Cell Ranger against the human reference genome to produce gene‐by‐cell UMI count matrices and per‐cell V(D)J contig annotations. Standard single‐cell quality control was applied at the cell level (library complexity, mitochondrial RNA fraction) and molecule level (valid barcodes and UMIs). The resulting matrices were used as inputs to downstream Seurat workflows, and contig annotation tables were retained for repertoire integration.

### 2.13 Normalization, Integration, and Annotation of scRNA‐seq Data

Gene‐count matrices were imported into the Seurat package. Mitochondrial fractions were computed from mitochondrial gene prefixes and used for routine filtering. Highly variable features were identified with the DUBStepR package. The integrated object was scaled, reduced by principal component analysis (PCA), and batch‐corrected with Harmony using batch labels to align shared biological structure across runs. The effective dimensionality (number of principal components retained) was determined by elbow plots of explained variance. A k‐nearest‐neighbor (kNN) graph was constructed in the Harmony space, communities were detected with the Louvain algorithm, and Uniform Manifold Approximation and Projection (UMAP) was computed from the same embedding to visualize global structure. Cluster identities were assigned to major immune and non‐immune lineages (epithelial, neutrophils, myeloid, T, B, and NK cells) by comparing cluster‐specific markers to canonical markers.

### 2.14 T Cell Subclustering Analysis

T‐cell clusters were subset from the integrated object and re‐embedded using the Harmony‐aligned dimensions. Subclusters were annotated using marker patterns consistent with functional states: PD‐1^−^CD4^+^T cells, PD‐1^+^CD8^+^T cells, regulatory T cells (Treg), PD‐1^+^CD4^+^T cells, and PD‐1^−^CD8^+^T cells. A compact marker panel (CD3D/E, CD4, FOXP3, PDCD1, IFNG, HAVCR2, LAG3, GZMB, TCF7, LEF1) was visualized with dot plots to document lineage and state assignments. The scRNA‐seq data were also used to distinguish progenitor‐exhausted T cells by TCF‐1 expression and terminally exhausted T cells by high TOX expression and positivity for CD39 and TIM‐3.

### 2.15 Differential Gene‐Expression Analysis

Differential gene expression analysis was performed to identify genes that were significantly upregulated in PD‐1^+^CD8^+^T cells compared to their PD‐1^−^ counterparts. The corresponding gene symbols were subsequently annotated with Entrez Gene IDs using the org.Hs.eg.db database. Separate over‐representation analyses were then carried out for Gene Ontology (GO) Biological Process terms, as well as Reactome pathways, utilizing the clusterProfiler and ReactomePA packages, respectively.

### 2.16 Pathway Module Scoring in CD8^+^T Cells

Within the T‐cell compartment, CD8^+^T‐cell subsets (PD‐1^−^ vs PD‐1^+^) were isolated. Curated gene sets representing IL‐6/JAK‐STAT3 (Hallmark) signaling were scored using Seurat's gene‐set scoring to quantify pathway activity at single‐cell resolution. Subset differences were summarized with violin plots supplemented by statistical annotations to indicate significance.

### 2.17 ScTCR‐seq Analysis

For each cell, TCR sequences were parsed from Cell Ranger V(D)J outputs. Only productive, in‐frame α/β rearrangements were retained when assigning a cell's dominant receptor. A clonotype was defined by the unique dominant α–β pairing; cells sharing the same α and β sequences were assigned to the same clonotype. Clonality was summarized by clonotype size (number of cells carrying that α‐β pair). Contigs were restricted to cells present in the CD8^+^ Seurat object (PD‐1^−^ and PD‐1^+^). Clonotypes were assembled with scRepertoire.

### 2.18 Clonal Architecture, CDR3 Length, and V‐J Usage

Clonal size classes and CDR3 length distributions were summarized from both amino‐acid and nucleotide sequences, jointly (both chains) and per chain (TRA/TRB). To visualize dominance and sharing of expanded clones, the largest clonotypes were compared between PD‐1^−^ and PD‐1^+^CD8^+^T cells using alluvial diagrams. Variable‐joining gene‐pairing matrices (TRBV‐TRBJ and TRAV‐TRAJ) were computed and displayed as heatmaps.

### 2.19 Diversity Analysis

Repertoire homeostasis and clonal proportions were summarized to assess the contribution of expanded versus rare clonotypes. Clonal occupancy was evaluated across cell identities. Diversity was then quantified with complementary indices (Shannon, inverse Simpson, Gini, ACE), capturing richness and evenness of the CD8^+^ repertoires and mitigating dependence on any single metric.

### 2.20 CDR3 K‐mer Profiling and Motif Analysis

CDR3α/β amino‐acid sequences were parsed per barcode. Short k‐mer compositions were profiled with immunarch (typical k in the 3‐5 range; parameters matched those used in the analysis) to highlight group‐specific amino‐acid patterns. For each chain and group, self‐similarity profiles and sequence logos were generated, revealing enriched motifs in PD‐1^+^ versus PD‐1^−^CD8^+^ cells.

### 2.21 GLIPH2 Analysis

TCR convergence was analyzed using the GLIPH2 algorithm implemented in the immGLIPH package. TRB CDR3 amino acid sequences from PD‐1^−^ and PD‐1^+^CD8^+^T cells were grouped according to local motif enrichment and global sequence similarity. Redundant motifs with identical cellular membership were collapsed into a single convergence group. For each group, enrichment between the two CD8^+^T‐cell subsets was assessed using a two‐sided Fisher's exact test, with TRB‐positive cells in each subset used as the denominator. *P* values were adjusted for multiple comparisons using the Benjamini‐Hochberg method. TCR similarity networks were generated based on GLIPH2‐inferred local and global relationships, with node color representing the GLIPH2 total score.

### 2.22 Statistical Analysis

All statistical analyses were two‐sided tests performed at a significance level of α = 0.05 (*p*<0.05). Differences in mean ± standard error of the mean (SEM) were evaluated with Student's t‐test (for normally distributed data), paired t‐test (for paired samples), and Welch's t‐test (when variances were unequal). Normality and homogeneity of variance were assessed using the Shapiro–Wilk test and F‐test, respectively. The correlation analysis was evaluated with Pearson correlation and linear regression, with the correlation coefficient (r) and coefficient of determination (R^2^) reported. The association between response status and TIL frequency (dichotomized as high/low based on median values) was analyzed using Fisher's exact test. Receiver operating characteristic (ROC) curves for response status were constructed to assess the prognostic ability of TILs, and the area under the curve (AUC) was calculated with corresponding 95% confidence intervals. The optimal cut‐off value was determined using the Youden Index (sensitivity+specificity‐1). All statistical analyses were performed using GraphPad Prism 8.0.

## 3 Results

### 3.1 Single‐Cell Transcriptional Profiles of TME From Patients with NSCLC

**FIGURE 1:**
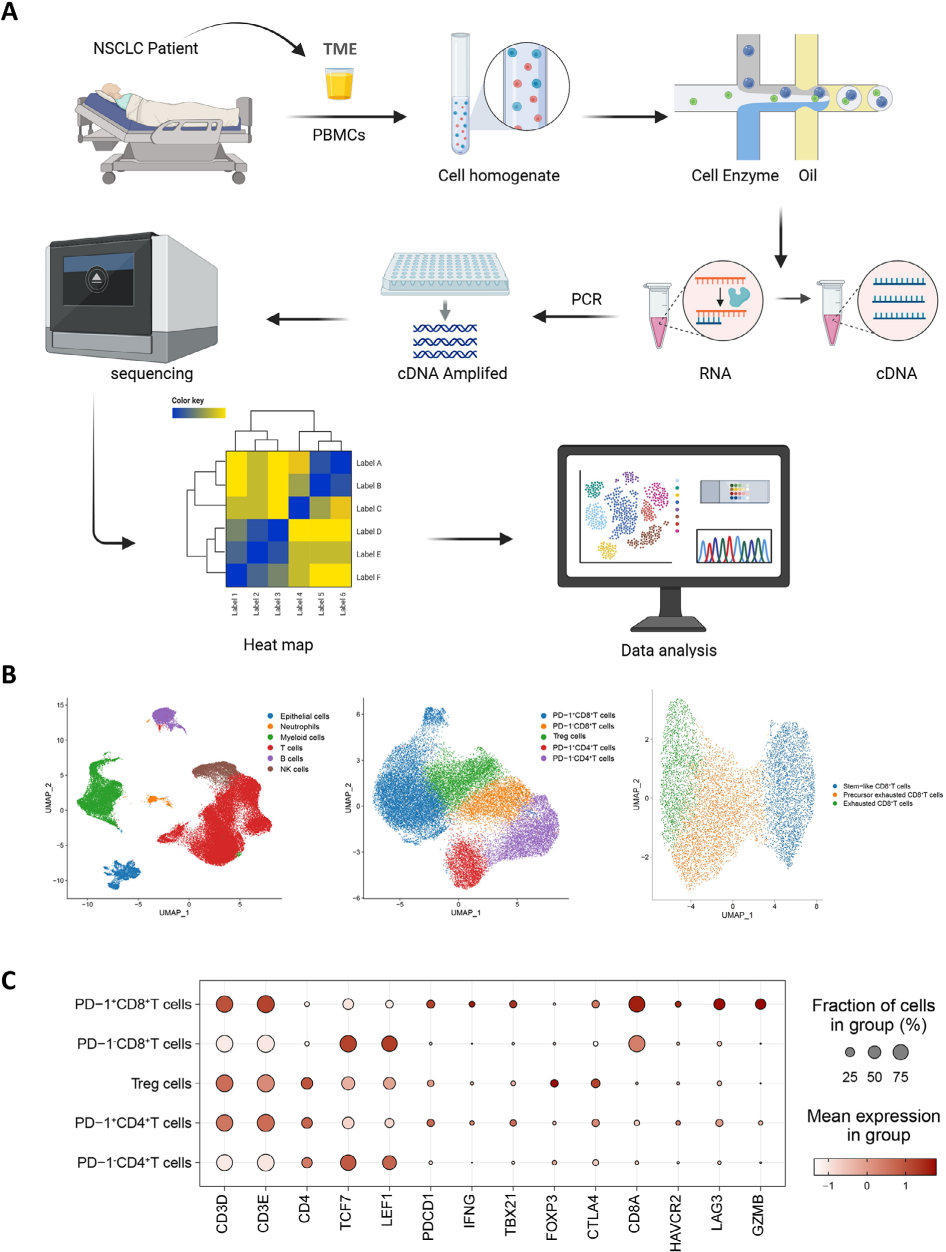
scRNA‐seq reveals diverse cell types in NSCLC from seven donors. (A) Flowchart for scRNA‐seq. (B) UMAP (left) of all single cells annotated by major lineages (each dot = one cell; color indicates cell type). UMAP (right) of the T cell subsets (PD‐1^−^CD8^+^T cells, PD‐1^+^CD8^+^T cells, Treg cells, PD‐1^−^CD4^+^T cells, PD‐1^+^CD4^+^T cells; stem‐like CD8^+^T cells, precursor exhausted CD8^+^T cells, exhausted CD8^+^T cells). (C) Dot plot of canonical T‐cell marker genes across T cell subsets; dot size denotes the fraction of cells expressing each gene and color indicates the mean (scaled) expression within the cluster.

To delineate the transcriptional programs associated with NSCLC progression, we performed scRNA‐seq using the 10x Genomics platform on tumor specimens from seven patients (Figure 1A). Unsupervised clustering combined with canonical marker genes identified six major cellular lineages within the TME‐epithelial cells, neutrophils, myeloid cells, T cells, B cells, and natural killer (NK) cells, which occupied clearly separated regions in the UMAP embedding. Focusing on the immune compartment, T cells were further partitioned into five transcriptionally and functionally distinct subsets: PD‐1^+^ and PD‐1^−^CD8^+^T cells, PD‐1^+^ and PD‐1^−^CD4^+^T cells, and regulatory T (Treg) cells. Additionally, it was also defined as stem‐like CD8^+^T cells, precursor exhausted CD8^+^T cells and exhausted CD8^+^T cells by TCF‐1, TOX expression, CD39 and TIM‐3 (Figure 1B). Differential gene‐expression analysis across these subsets revealed pronounced transcriptional divergence, particularly between PD‐1^+^ and PD‐1^−^ CD8^+^T cells (Figure 1C). Collectively, these findings suggest that an increased abundance of PD‐1^+^CD8^+^T cells may define a more aggressive biological subtype of NSCLC.

### 3.2 Characterization of State of PD‐1^+^/^−^CD8^+^T Cells by scRNA‐Seq

To further define the functional programs associated with PD‐1^+^CD8^+^T cells, we performed GO Biological Process and Reactome pathway enrichment analyses on genes upregulated in PD‐1^+^ versus PD‐1^−^CD8^+^T cells. Detailed, GO analysis revealed a strong enrichment of terms related to T cell activation and effector function, including immune response activating signaling pathway, T cell mediated immunity, leukocyte mediated cytotoxicity, T cell and leukocyte proliferation, leukocyte migration, lymphocyte differentiation, and antigen processing and presentation/peptide antigen assembly with MHC protein complex, as well as multiple categories linked to positive regulation of cell and leukocyte cell‐cell adhesion and actin‐filament organization (Figure S1A–D). Reactome analysis yielded concordant results, highlighting pathways such as neutrophil degranulation, signaling by interleukins, TCR signaling and downstream TCR signaling, regulation of T‐cell activation by the CD28 family, phosphorylation of CD3 and TCR ζ chains, translocation of ZAP‐70 to the immunological synapse, co‐inhibition by PD‐1, RHO GTPase cycle/effectors, platelet activation and aggregation, and programmed cell death (Figure S1E–H). Collectively, these enrichment patterns indicate that PD‐1^+^CD8^+^T cells in NSCLC are transcriptionally impaired and biased toward antigen‐driven activation, cytotoxic effector responses, and dynamic regulation of TCR and co‐receptor signaling within the TME.

### 3.3 Skewed TCR Vβ Repertoire Usage in PD‐1^+^CD8^+^T Cells

This has led to the hypothesis that the generation and evolution of the TCR repertoire are impacted in this T cell population. Then, we quantified 24 TCR‐Vβ subfamilies in tumor samples from 19 patients, and found 17 to be significantly enriched in PD‐1^+^CD8^+^T cells relative to PD‐1^−^CD8^+^T cells, indicating pronounced clonal expansion and skewed TCR‐Vβ usage in the PD‐1^+^ compartment (Figure S2). In detail, 17 families showed preferential enrichment, while the remaining 7 exhibited comparable usage between subsets. Specifically, Vβ5.3, Vβ7.1, Vβ3, Vβ9, Vβ16, Vβ18, Vβ5.1, Vβ20, Vβ13.1, Vβ8, Vβ5.2, Vβ2, Vβ12, Vβ23, Vβ1, Vβ11, and Vβ13.2 were significantly enriched within PD‐1^+^CD8^+^T cells, whereas Vβ17, Vβ13.6, Vβ21.3, Vβ22, Vβ14, Vβ4, and Vβ7.2 indicated no differences in the two groups. These findings indicate that PD‐1^+^CD8^+^T cells display significantly reduced clonal diversity, which may indicate a possible attenuation of immune response upon anti‐PD‐1 therapy.

### 3.4 TCR Repertoire Analysis Appears to be Mutually Exclusive in PD‐1^+^ versus PD‐1^−^CD8^+^T Cells

**FIGURE 2:**
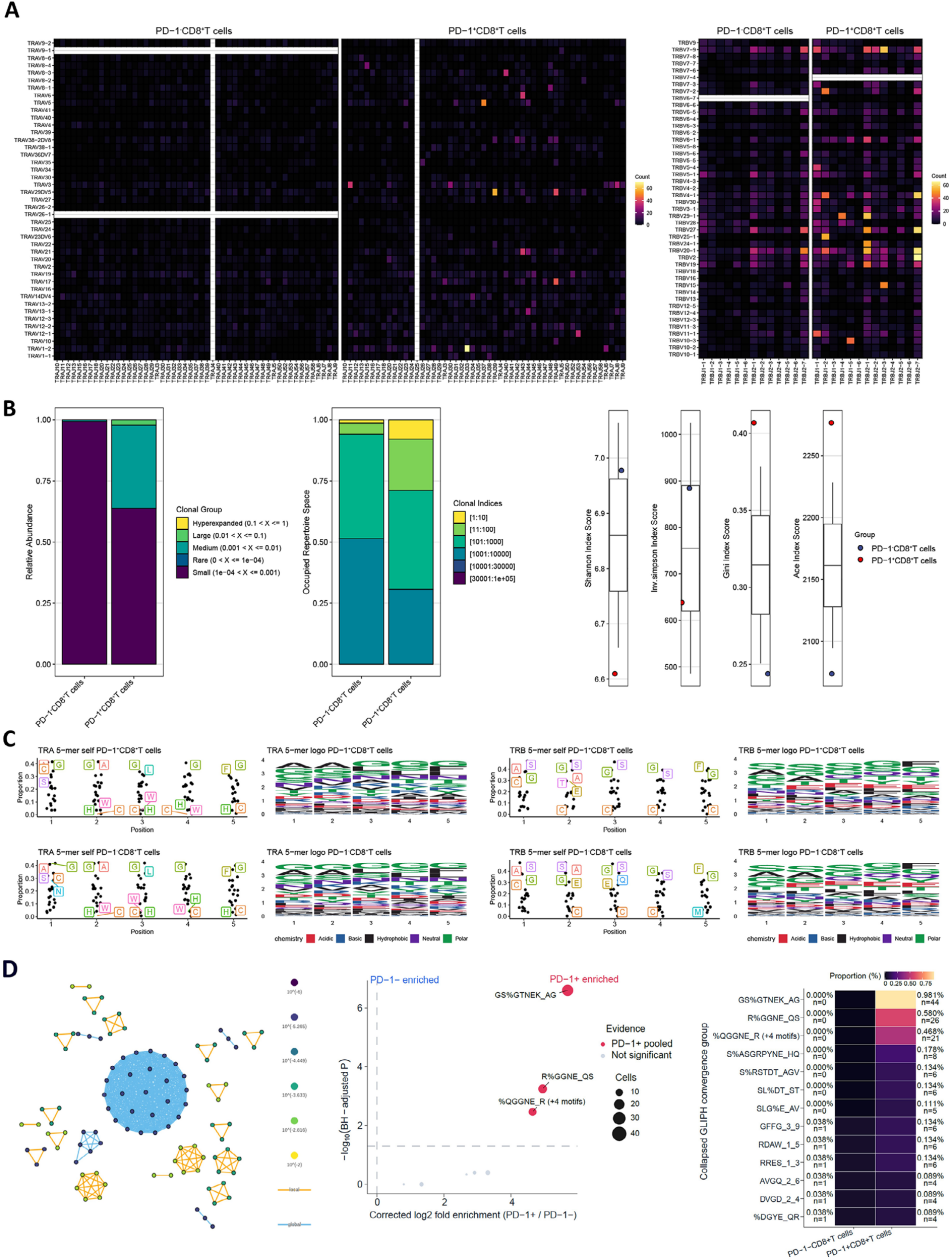
TCR immunome analysis of PD‐1^+^ vs. PD‐1^−^ CD8^+^T cells. (A) V‐J gene rearrangement usage heatmaps. TRA (left) and TRB (right) V‐J pairing heatmaps for PD‐1^−^CD8^+^T cells and PD‐1^+^CD8^+^T cells. The y‐axis lists V genes and the x‐axis lists J genes. Color intensity indicates the usage level (counts/normalized usage) of each V‐J combination within the corresponding subset, revealing distinct preferential V‐J pairing patterns between PD‐1^−^ and PD‐1^+^ repertoires. (B) Clonal expansion profile and diversity metrics. Left, relative abundance of clonotypes stratified by expansion tiers: Hyperexpanded (0.1 < X ≤ 1), Large (0.01 < X ≤ 0.1), Medium (0.001 < X ≤ 0.01), Small (1×10^−4^ < X ≤ 0.001), and Rare (0 < X ≤ 1×10^−4^), where X denotes clonotype frequency. Middle, repertoire space contribution grouped by clonotype rank (clonal index bins; e.g., 1‐10, 11‐100, 101‐1,000, 1,001‐10,000, 10,001‐100,000, 100,001‐10^6^). Right, boxplots comparing repertoire diversity/inequality estimators (Shannon, inverse Simpson, Gini, and ACE) between PD‐1^−^ and PD‐1^+^ subsets. (C) CDR3 5‐mer sequence features. TRA and TRB CDR3 amino‐acid 5‐mer frequency spectra (left panels) and corresponding sequence logo plots (right panels) for PD‐1^−^ and PD‐1^+^CD8^+^T cells. Logos highlight positional amino‐acid preferences; residues are colored by physicochemical class (acidic, basic, hydrophobic, neutral, polar), indicating divergent antigen‐recognition motifs and structural constraints between the two subsets. (D) Network of GLIPH2‐defined TCR convergence groups. Nodes represent unique CDR3β sequences, and edges indicate local or global sequence similarity. Node color reflects the GLIPH2 total score. Differential enrichment of collapsed GLIPH2 convergence groups between PD‐1^−^ and PD‐1^+^CD8^+^T cells. The x‐axis shows corrected log_2_fold enrichment, the y‐axis shows −log_10_ (BH‐adjusted *P*), and point size indicates cell number. Relative abundance of collapsed GLIPH2 convergence groups in the two subsets. Tile color indicates cell proportion, with percentages and cell counts shown within each tile.

Antigen‐specific T cells are central effectors of antitumor immunity, and TCR repertoire profiling provides a robust surrogate readout of tumor antigen‐driven responses. We therefore characterized the intratumoral TCR repertoire in NSCLC to compare PD‐1^+^ and PD‐1^−^CD8^+^T cell subsets. Reconstruction of paired TCR chains revealed that PD‐1^+^ and PD‐1^−^CD8^+^T cells harbored largely non‐overlapping clonotypes and exhibited distinct V‐J gene usage and V‐J pairing patterns (Figure 2A–C and Figure S3). Their clonal architectures also diverged: PD‐1^−^CD8^+^T cells were dominated by rare and small clones, whereas PD‐1^+^CD8^+^T cells contained a higher proportion of medium‐to‐large and hyperexpanded clones, resulting in a more even distribution across clone‐size categories. In line with these findings, repertoire diversity metrics‐including the Shannon and inverse Simpson indices, Gini coefficient, and ACE estimator‐demonstrated greater clonal diversity in PD‐1^−^CD8^+^T cells and a more oligoclonal, uneven repertoire among PD‐1^+^CD8^+^T cells. Moreover, analysis of 5‐mer amino acid motifs within TRA and TRB CDR3 regions revealed distinct compositional and frequency profiles between the two subsets. Sequence logo representations highlighted differences in positional amino acid conservation and physicochemical properties, suggesting divergent antigen‐recognition signatures and structural constraints that may underlie their functional specialization. Furthermore, GLIPH2 analysis revealed distinct TCR convergence groups within the CD8^+^T cell compartment (Figure 2D). Following consolidation of motifs with identical or highly overlapping cellular membership, GS%GTNEK_AG, R%GGNE_QS, and %QGGNE_R (+4 motifs) were significantly enriched in PD‐1^+^CD8^+^T cells. These findings supported selective expansion of TCRs with convergent sequence features in the PD‐1^+^ subset, potentially reflecting immune responses directed against shared or structurally related antigenic determinants.

### 3.5 Analysis of Tumoral PD‐1^+^CD8^+^T Cells in NSCLC

To further evaluate the clinical relevance of PD‐1^+^CD8^+^T cells, we assessed their presence in the TME of treatment‐naïve NSCLC patients (Figure S4A). The baseline characteristics of the 105 enrolled NSCLC patients are summarized in Table 1. The cohort had a mean age of 67.38 years (range: 37‐87 years), and 82.86% of the patients were male. According to histological classification, 69 cases were squamous cell carcinoma and 36 were adenocarcinoma. More than half (56.19%) of the patients were diagnosed at an advanced pathological stage (IIIB‐IV), and 64.76% (68 patients) had a primary tumor greater than 3 cm in size.

**TABLE 1:** Patient clinical characteristics.

Characteristics	Number (%)	
Sex		
Male	87	(82.86)
Female	18	(17.14)
Age (years)		
< 65	35	(33.33)
≥ 65	70	(66.67)
Size(cm)		
< 3	37	(35.24)
≥ 3	68	(64.76)
Stage		
≤IIIA	46	(43.81)
>IIIA	59	(56.19)
Types		
NSCLC	105	(100)
Adenocarcinomas	36	(34.29)
Squamous carcinomas	69	(65.71)
TME		
Non‐central	79	(75.24)
Central	26	(24.76)

Analysis of PD‐1^+^CD8^+^T‐cell infiltration revealed significantly higher levels in male patients compared with female patients (48.80% ± 25.30% vs. 31.94% ± 23.64%, p < 0.05), in older patients (50.15% ± 25.32% vs. 37.42% ± 24.72%, p < 0.05), and in those with advanced‐stage disease (52.51% ± 26.73% vs. 41.78% ± 21.96%, *p*<0.05). In contrast, no significant difference in PD‐1^+^CD8^+^T cell infiltration was observed between patients with larger (≥3 cm) and smaller (<3 cm) primary tumors (48.05% ± 23.46% vs. 41.96% ± 29.36%, *p*>0.05) (Figure S4B).

### 3.6 PD‐1^+^CD8^+^T Cells were Spatially Accumulated in the Non‐Central Zone of the TME

**FIGURE 3:**
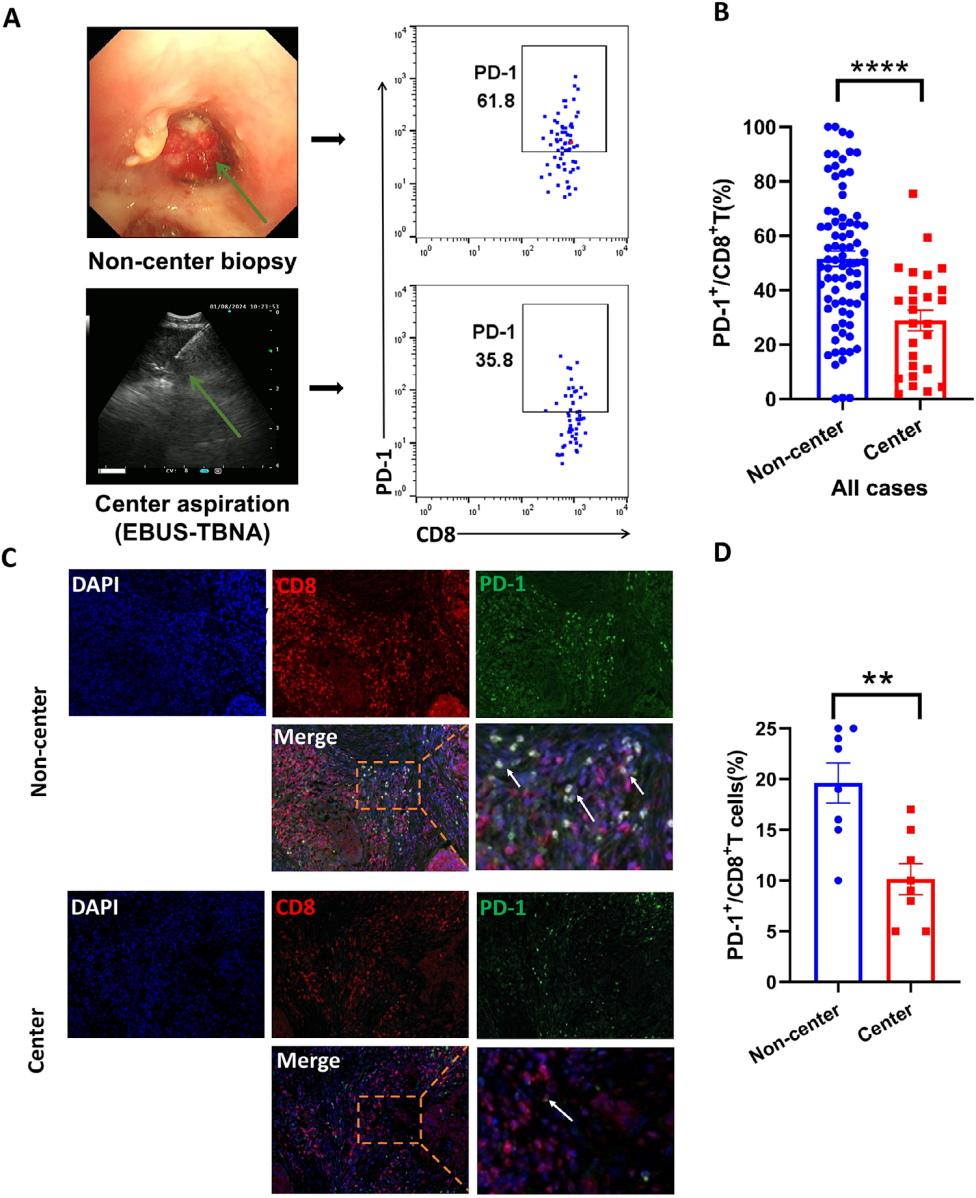
Regional characteristics of PD‐1^+^CD8^+^T cell infiltration in the TME of NSCLC. (A) Tissue specimens from non‐central and central tumor regions were obtained by superficial biopsy and endobronchial ultrasound‐guided transbronchial needle aspiration (EBUS‐TBNA), respectively. (B) Quantitative analysis showing that the frequency of PD‐1^+^CD8^+^T cells was significantly higher in the non‐central tumor region than in the central tumor region (*n* = 105). (C,D) Representative immunofluorescence images (200× magnification) illustrating the co‐localization of CD8 (red) and PD‐1 (green) in non‐central versus central tumor regions (*n*=8). Each dot represents the mean value derived from three fields per case. Data are presented as mean±SD. Statistical significance was determined by unpaired Student's t‐test. **p < 0.01, ****p < 0.0001; ns: not significant.

Given the complex and regionally distinct phenotype of the NSCLC tumor microenvironment, the spatial heterogeneity of T cells requires further investigation. To evaluate the distribution of PD‐1^+^CD8^+^T cells across different tumor regions, we identified this T cell subset in both non‐central and central regions of the TME using spatially guided biopsy techniques (Figure 3A). As shown in Figure 3B, the proportion of PD‐1^+^CD8^+^T cells among total CD8^+^T cells was significantly higher in the non‐central TME (51.51% ± 25.19%) compared to the central TME (28.87% ± 19.26%; *p*<0.0001). To further validate and visualize the heterogeneous infiltration of PD‐1^+^CD8^+^T cells, immunofluorescence staining was performed on serial tissue sections from NSCLC samples. Consistent with the flow cytometry data, PD‐1^+^CD8^+^T cells exhibited a spatially distinct infiltration pattern, with greater density observed in the non‐central regions (Figure 3C,D). These findings provide preliminary evidence that the abundance of PD‐1^+^CD8^+^T cells varies regionally within the NSCLC TME, potentially contributing to immune heterogeneity.

### 3.7 Abundance of Non‐Central PD‐1^+^CD8^+^T Cells Predicted Inferior Efficacy of PD‐1 Blockade

**FIGURE 4:**
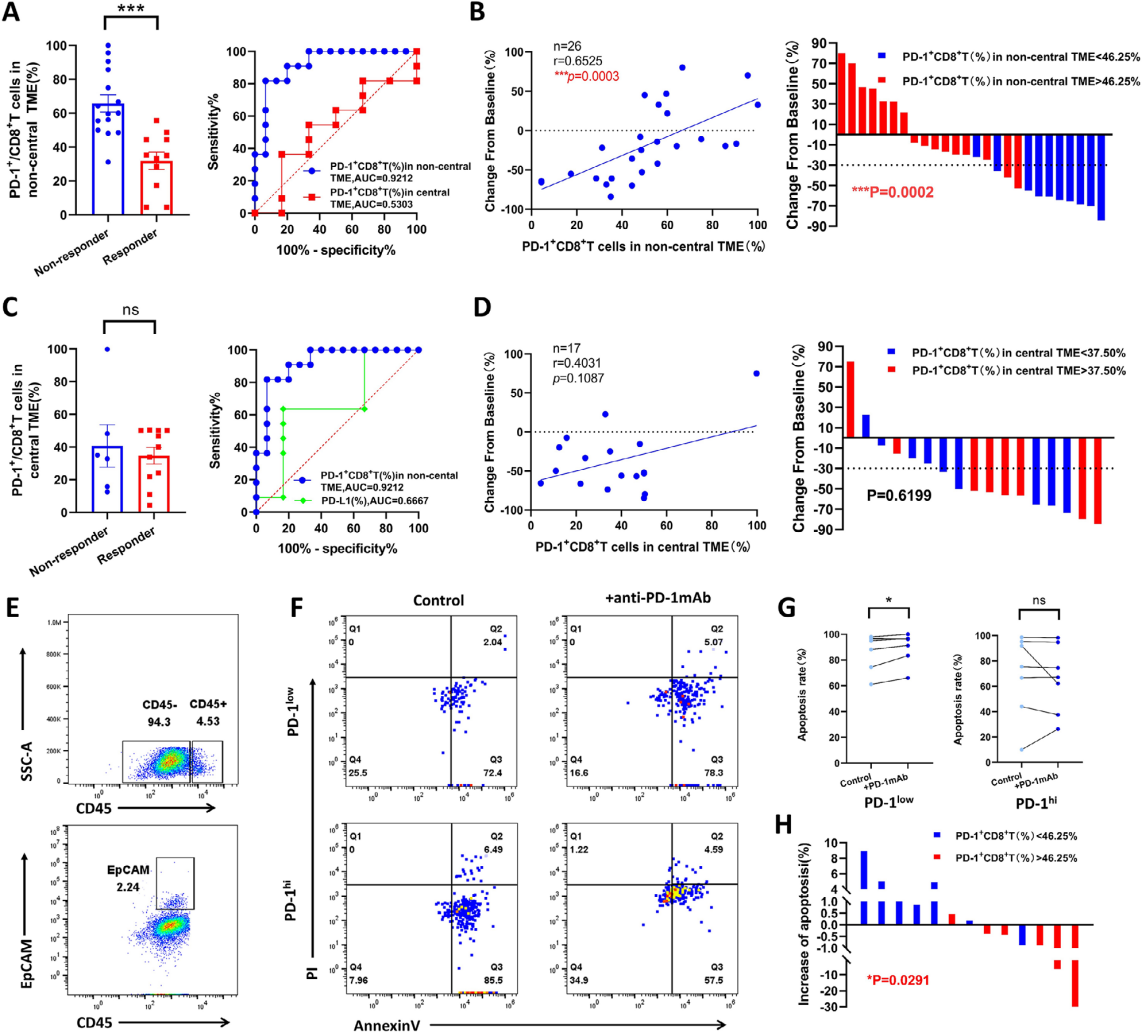
Analysis of PD‐1^+^CD8^+^T cells in NSCLC to predict the efficacy of anti‐PD‐1 mAb. (A,B) PD‐1^+^CD8^+^T cells localized in the non‐central regions of tumor tissues. (C, D) PD‐1^+^CD8^+^T cells localized in the central regions of tumor tissues. A receiver operating characteristic (ROC) curve was constructed to assess the predictive value of PD‐1^+^CD8^+^T cell infiltration levels for anti‐PD‐1 therapeutic efficacy. The correlation between PD‐1^+^CD8^+^T cell infiltration levels and the extent of tumor regression following anti‐PD‐1 treatment was analyzed using Pearson correlation analysis. Tumor response was visualized by a waterfall plot based on the percentage change in the maximum diameter of target lesions. (E) Gating strategy for identifying tumor cells in fresh resected NSCLC tissues by flow cytometry. (F) Representative flow cytometric analysis of tumor cell apoptosis after 72 h anti‐PD‐1 mAb treatment. (G,H) Quantitative analysis of tumor cell apoptosis stratified by baseline PD‐1^+^CD8^+^T cell infiltration levels in the tumor microenvironment following anti‐PD‐1 antibody therapy. Data are presented as the mean±SD. Statistical significance was determined by unpaired Student's t‐test or paired Student's t‐test. *p < 0.05, ***p < 0.001; ns: not significant.

We next investigated whether PD‐1^+^CD8^+^T cells in different tumor regions could serve as predictive biomarkers for response to anti‐PD‐1 mAb therapy. Among the 43 patients who received anti‐PD‐1 mAb therapy, responders exhibited significantly lower non‐central infiltration of PD‐1^+^CD8^+^T cells compared to non‐responders (31.96±17.10% vs. 65.79±19.79%, *p* < 0.001, Figure 4A). The optimal cutoff value for predicting ORR based on non‐central PD‐1^+^CD8^+^T cell infiltration was 46.25%, yielding high accuracy (88.46%), specificity (93.33%), sensitivity (81.82%), and robust diagnostic performance (AUC = 0.9212). Moreover, lower infiltration of PD‐1^+^CD8^+^T cells in the non‐central TME was associated with greater clinical regression following anti‐PD‐1 mAb therapy (Figure 4B). In contrast, PD‐1^+^CD8^+^T cells located in the central TME did not demonstrate predictive value for anti‐PD‐1 mAb therapy (Figure 4C,D). Specifically, using a cutoff of 37.50%, central PD‐1^+^CD8^+^T cells achieved only 58.82% accuracy, 66.67% specificity, 54.55% sensitivity, and an AUC of 0.5303. Collectively, these findings suggest that the proportion of PD‐1^+^CD8^+^T cells in the non‐central TME better reflects the functional characteristics of CD8^+^T cells responsive to PD‐1 blockade.

### 3.8 Anti‐PD‐1 Therapy Promoted Lung Cancer Cell Apoptosis In Vitro Dependent on TME Shaped by PD‐1^+^CD8^+^T Cells

To evaluate whether immune checkpoint blockade response is impaired in patients with higher PD‐1^+^CD8^+^T cell infiltration, single‐cell suspensions derived from human NSCLC tissues were treated with anti‐PD‐1 mAb in vitro for 72 h to simulate therapeutic conditions. An analytical protocol was established to identify viable lung cancer cells and assess apoptosis following anti‐PD‐1 mAb treatment (Figure 4E,F). Based on PD‐1^+^CD8^+^T cell infiltration levels, 13 patients were stratified into high‐ and low‐infiltration subgroups. As shown in Figure 4G, anti‐PD‐1 treatment significantly increased apoptosis of fresh lung cancer cells in the low‐infiltration subgroup (*p* < 0.05), whereas no significant change was observed in the high‐infiltration subgroup (*p* > 0.05). Consistently, a greater extent of anti‐PD‐1‐induced cancer cell apoptosis was detected in patients with lower PD‐1^+^CD8^+^T cell infiltration (*p* < 0.05, Figure 4H). These results indicate that the efficacy of PD‐1 blockade in promoting cancer cell apoptosis is inversely correlated with PD‐1^+^CD8^+^T cell infiltration levels, suggesting that a dysfunctional phenotype of these cells may contribute to reduced therapeutic responsiveness.

### 3.9 Involvement of BTLA^+^B Cells in Inducing Spatial Distribution of Tumoral PD‐1^+^CD8^+^T Cells

**FIGURE 5:**
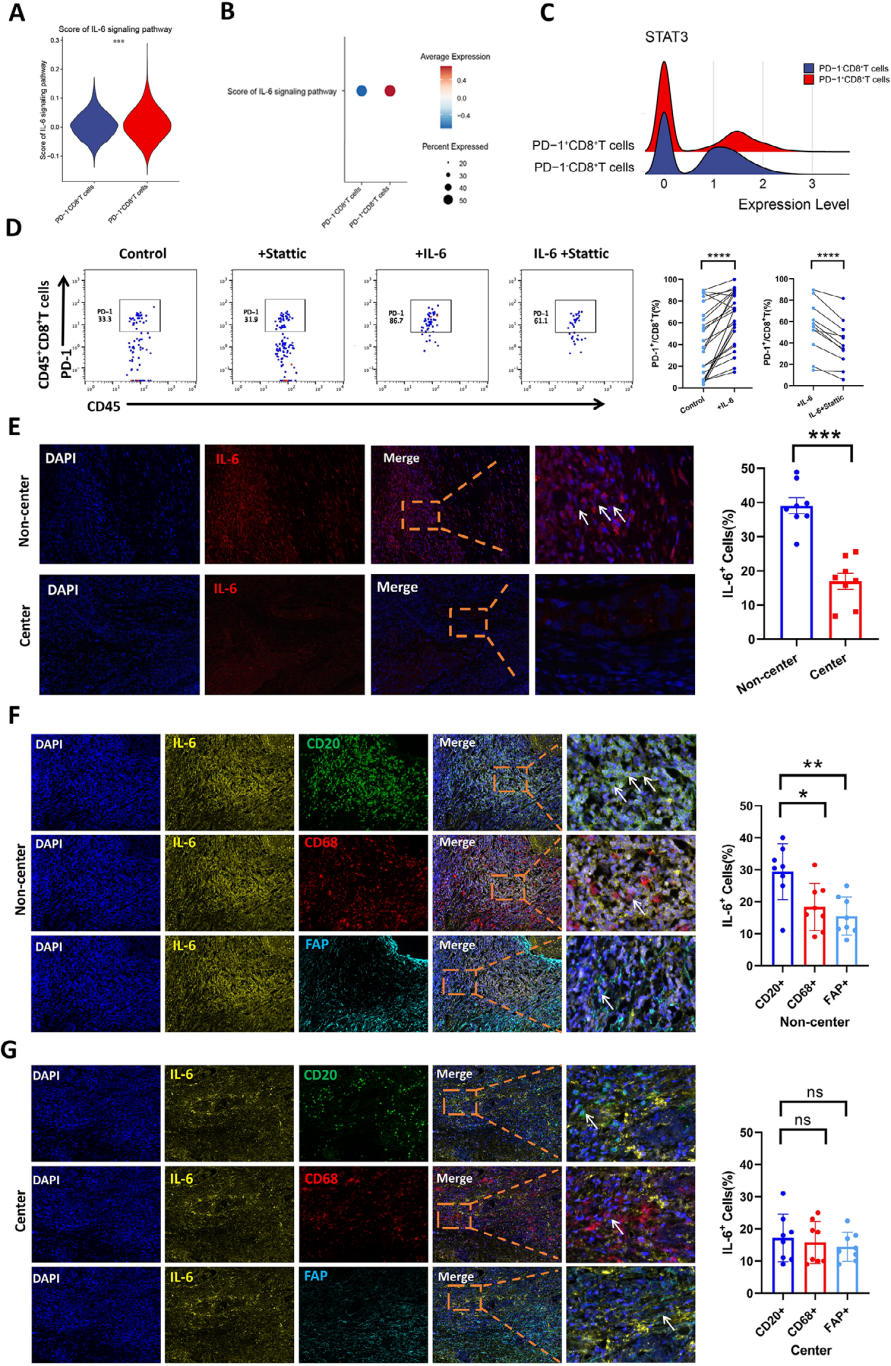
IL‐6 promotes the expression of PD‐1 on CD8^+^T cells derived from lung cancer. (A) Pathway activity scores for IL‐6 signaling in PD‐1^+^CD8^+^T cells compared with PD‐1^−^CD8^+^T cells. (B) Dot plot displaying core IL‐6 pathway genes across the two CD8^+^T cell subsets; dot size represents the fraction of gene‐expressing cells, and color intensity reflects mean (scaled) gene expression. Both parameters are elevated in PD‐1^+^CD8^+^T cells relative to PD‐1^−^CD8^+^T cells. (C) Differential expression of STAT3 between groups, with higher levels observed in PD‐1^+^CD8^+^T cells. (D) Recombinant IL‐6 significantly upregulated PD‐1 surface expression on CD8^+^T cells via the STAT3 pathway (*n* = 25 for Control vs. IL‐6; *n* = 11 for IL‐6 vs. IL‐6 + Stattic). (E) Representative immunofluorescence images of IL‐6 in NSCLC tissue sections (200× magnification) (*n* = 8). (F,G) Multiplex immunofluorescence staining for CD20 (B cells), CD68 (macrophages), FAP (cancer‐associated fibroblasts), and IL‐6 in central and non‐central tumor regions from the same tissue sections. Representative images show immunofluorescence co‐localization (200× magnification) (*n* = 8). Each dot represents the mean value derived from three fields per case. Data are presented as the mean±SD. Statistical significance was determined by unpaired Student's t‐test or paired Student's t‐test. *p < 0.05, **p < 0.01, ***p < 0.001; ns: not significant.

To explore the intrinsic mechanisms governing the spatial organization of PD‐1^+^CD8^+^T cell subsets within the TME, we analyzed the association between the PD‐1^+^CD8^+^ T cell transcriptional signature and potentially targetable cytokines. scRNA‐seq analysis revealed that PD‐1^+^CD8^+^T cells had significantly higher pathway activity scores for IL‐6 signaling compared to PD‐1^−^CD8^+^T cells (Figure 5A,B). Furthermore, Ingenuity Pathway Analysis (IPA) indicated differential activation of STAT3 between PD‐1^−^ and PD‐1^+^CD8^+^T cell subsets, consistent with active IL‐6 signaling, a known upstream regulator of PD‐1 expression (Figure 5C). Supporting these findings, IL‐6 stimulation significantly increased PD‐1 expression levels and elevated the proportion of PD‐1^+^CD8^+^T cells within the total CD8^+^T cell population, while inhibition of STAT3 signaling with Stattic partially reversed this IL‐6‐mediated upregulation of PD‐1 expression on CD8^+^T cells (Figure 5D).

**FIGURE 6:**
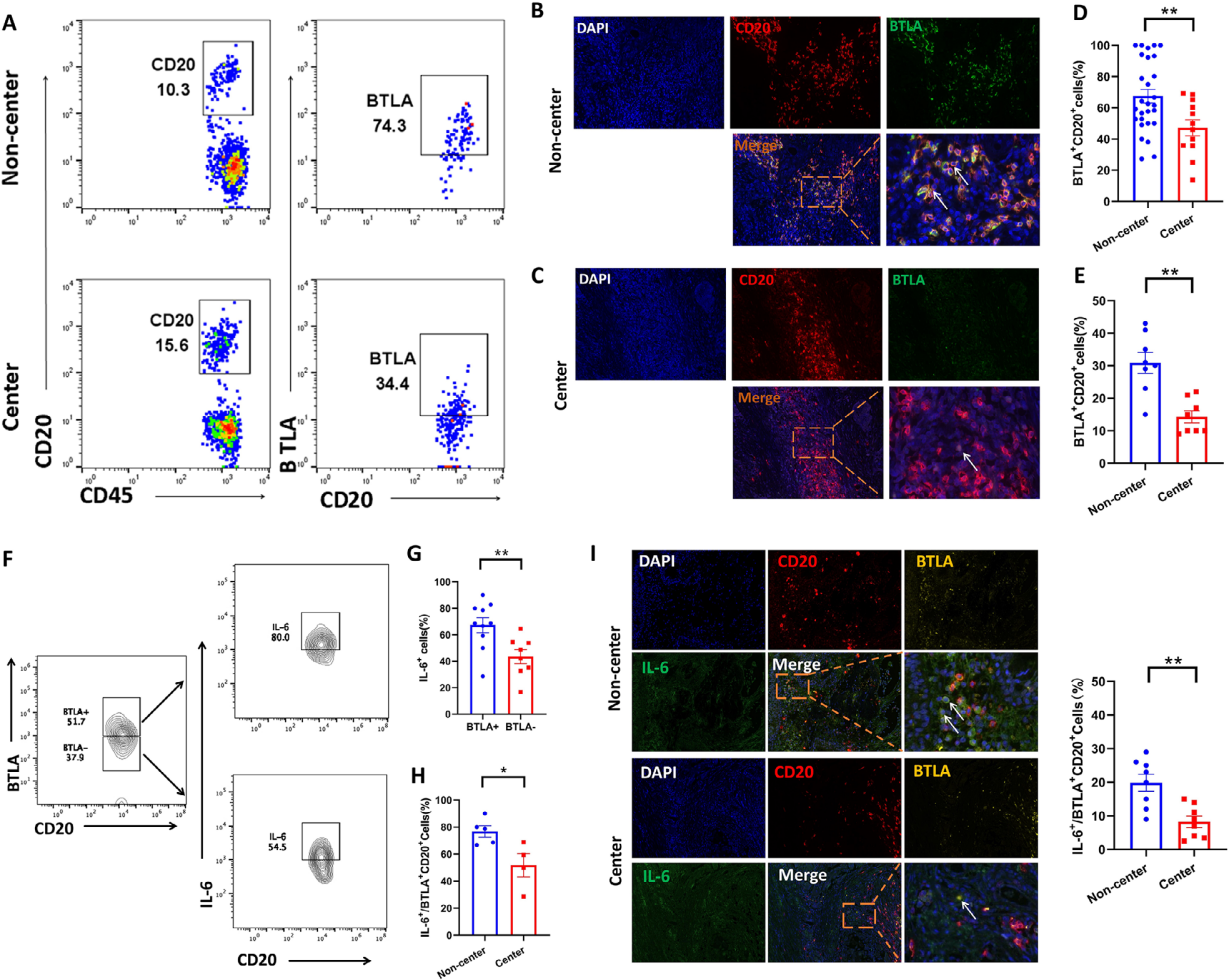
Infiltration of BTLA^+^CD20^+^B cells among NSCLC TME and its capacity to secrete IL‐6. (A) Representative flow cytometry density plots illustrate BTLA^+^CD20^+^B‐cell populations isolated from central versus non‐central tumor regions. (B,C) Immunofluorescence imaging (200× magnification) of the central and non‐central tumor regions, confirming the co‐localization of BTLA (green) and CD20 (red). (D,E) Spatial position analysis reflects more BTLA^+^CD20^+^B cell clustering within the non‐central region (D, *n* = 40; E, *n* = 8). (F–H) Comparative analysis of IL‐6 secretion was conducted between BTLA^−^CD20^+^ and BTLA^+^CD20^+^B cell subsets, and BTLA^+^CD20^+^B cells isolated from the central versus non‐central tumor regions (G, *n* = 12; H, *n* = 9). (I) Spatial IL‐6^+^BTLA^+^B cells were further visualized and quantified using multiplex immunofluorescence staining (representative images shown at 200× magnification) (*n* = 8). Each dot represents the mean value derived from three fields per case. Data are presented as mean±SD. Statistical significance was determined by unpaired Student's t‐test. *p < 0.05, **p < 0.01; ns: not significant.

Furthermore, the spatial distribution of IL‐6‐producing cells was assessed by immunofluorescence staining. Notably, non‐central tumor regions exhibited significantly higher enrichment of these cytokines compared to the tumor center (Figure 5E). Collectively, these data suggest that regionally produced IL‐6 within the TME may play a mechanistic role in driving the heterogeneous infiltration of PD‐1^+^CD8^+^T cells. Next, we sought to identify the cellular sources of IL‐6 within non‐central tumor regions. Current evidence indicates that tumor‐associated macrophages (TAMs) and cancer‐associated fibroblasts (CAFs) are major sources of IL‐6 in the NSCLC TME. Additionally, intratumoral B cells have been implicated in T cell recruitment and shown to be important for effective anti‐PD‐1 therapy. Initial multiplex immunofluorescence analysis revealed no significant differences in the proportions of B cells, TAMs, and CAFs among the total IL‐6‐secreting cell population in the tumor center. In contrast, within the non‐central tumor region, B cells were the predominant IL‐6‐secreting cell type (Figure 5F,G). Subsequently, FCM and immunofluorescence analyses were performed to delineate the in vivo spatial distribution of a specific B cell subset within the NSCLC TME (Figure 6A–E). Consistent with the previously observed distribution of PD‐1^+^CD8^+^T cells, BTLA^+^B cells were predominantly localized in non‐central tumor regions, whereas BTLA^−^B cells were more frequently detected in the central tumor area. To further validate these findings, among the total B cell population, the BTLA^+^ subset exhibited significantly greater IL‐6 secretion compared to the BTLA^−^ subset. Moreover, BTLA^+^B cells derived from non‐central regions produced higher levels of IL‐6 than those from the tumor center (Figure 6F‐H). Multiplex immunohistochemistry (mIHC) staining was also employed to detect IL‐6‐producing BTLA^+^B cells within the TME. Analysis revealed that these cytokine‐producing BTLA^+^B cells were spatially enriched in non‐central tumor regions compared to the central tumor (Figure 6I). Collectively, these findings delineate the phenotypic developmental trajectory of PD‐1^+^CD8^+^T cells and indicate that BTLA^+^B cells located in the non‐central region, rather than the tumor center, contribute to shaping this heterogeneous immune microenvironment in NSCLC.

### 3.10 Spatial Architecture Composed of PD‐1^+^CD8^+^T Cells and BTLA^+^B Cells

**FIGURE 7:**
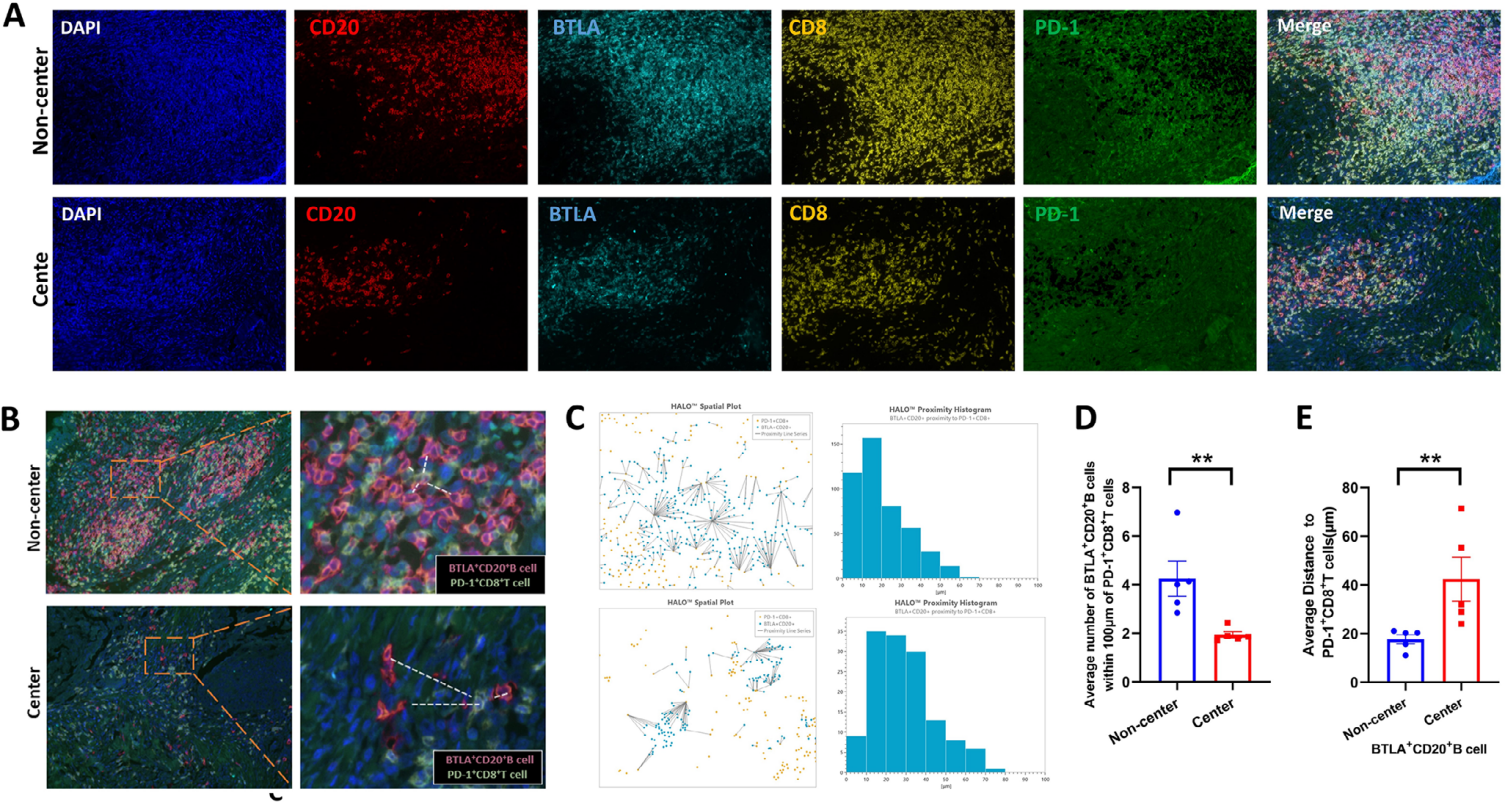
Spatial distribution of BTLA^+^CD20^+^B cells in NSCLC tissues and its correlation with PD‐1^+^CD8^+^T cells. (A) Representative multiplex immunofluorescence images (200× magnification) of NSCLC tissue sections showing DAPI (dark blue, nuclei), CD20 (red, B cells), BTLA (light blue), CD8 (yellow, T cells), and PD‐1 (green) in central and non‐central tumor regions. (B) Spatial distribution patterns of BTLA^+^CD20^+^B cells and PD‐1^+^CD8^+^T cells across distinct tumor regions (200× magnification). (C) Representative proximity plots and histograms depicting the number of BTLA^+^CD20^+^B cells within a 100 µm radius of PD‐1^+^CD8^+^T cells. (D, E) Quantitative analysis of the average number of PD‐1^+^CD8^+^T cells in proximity to BTLA^+^CD20^+^B cells and the mean intercellular distance between these immune cell subsets (*n* = 5). Statistical significance was determined by unpaired Student's t‐test. **p < 0.01.

To investigate the spatial organization between BTLA^+^B cells and PD‐1^+^CD8^+^T cells, we performed mIHC to characterize regional immune infiltrates. Representative multiplex fluorescence images show co‐staining for CD8 (yellow), CD20 (red), PD‐1 (green), BTLA (light blue), and DAPI (dark blue) in NSCLC tissue (Figure 7A). Compared to the central tumor region, non‐central areas exhibited increased infiltration of BTLA^+^B cells in close proximity to PD‐1^+^CD8^+^T cells, with a significantly reduced intercellular distance between these subsets (*p*<0.01, Figure 7B–E). These results indicate that BTLA^+^B cells and PD‐1^+^CD8^+^T cells form a closely associated cellular neighborhood in the non‐central tumor region, and their co‐enrichment within specific niches may reflect a distinct immune contexture that potentially contributes to immunotherapy resistance in NSCLC.

## 4 Discussion

The heterogeneity of the TME organized by various immune and stromal cells is a major contributing factor to tumor metastasis, relapse and anti‐PD‐1 resistance [17, 18], but how different TME subtypes are connected to clinical relevance in lung cancer remains unclear. Here, we developed analytical biopsy techniques to systematically characterize the TME of NSCLC and discovered that PD‐1^+^CD8^+^T cells selectively co‐localized with BTLA^+^B cells within the non‐central region of the tumor. This spatial niche offers insights into a possible link between immunotherapy resistance and regional immune profiles.

First, our study highlighted the spatial heterogeneity of PD‐1^+^CD8^+^T cells in NSCLC and profiled detailed cellular crosstalk. Studies have shown that TME can upregulate the expression level of PD‐1 on tumor‐specific CD8^+^T cells. Furthermore, studies [16] have found that the EMT/MSS subtype GC was reported to correlate with a worse prognosis and a high risk of recurrence, which may partly account for why PD‐1^+^CD8^+^T cell high‐infiltration tumors were extremely lethal. Therefore, it is crucial to identify the infiltration characteristics of PD‐1^+^CD8^+^T cells in NSCLC. Here, our research expanded this point, demonstrating that the PD‐1^+^CD8^+^T cell subsets selectively accumulated in the non‐central regions rather than the central regions of the tumor. Although it has been shown that anti‐PD‐1 can reinvigorate exhausted CD8^+^T cells [19, 20], our data suggested that it seems to be dependent on non‐central regions of the TME, where PD‐1^+^CD8^+^T cells served as a resistant biomarker for anti‐PD‐1 therapy and worked as a fundamental element of immunotherapy. Accordingly, we established that the PD‐1^+^CD8^+^T cell infiltrated conditions are the crucial determinant of anti‐PD‐1 therapeutic outcome. These data provide a potential explanation for some of the outcomes we see in the clinical setting since most cancer patients present with anti‐PD‐1 resistance. Of note, our cohort exhibits a marked male predominance, which may limit the generalizability of the findings across sexes. This imbalance is hypothesized to be related to the higher frequency of peripheral lung cancer occurrence in females, rendering lesions less accessible to real‐time bronchoscopic biopsy under direct visualization. Given that currently available public NSCLC spatial transcriptomics datasets with matched anti‐PD‐1 response data do not specify whether samples were derived from the tumor center or the non‐tumor boundary regions, our findings warrant confirmation through prospective validation studies.

Actually, PD‐1^+^CD8^+^T cells might also be a heterogeneous cell subset with functional diversity. Previously, it was found that intratumoral Tcf1^+^PD‐1^+^CD8^+^T cells with stem‐like properties promote tumor control in response to vaccination and checkpoint blockade immunotherapy [21]. PD‐1 blockade in unprimed or suboptimally primed CD8 cells induces resistance through the induction of PD‐1^+^CD38^hi^CD8^+^T cells, who serve as a predictive and therapeutic biomarker for anti‐PD‐1 treatment [22]. Given our finding that PD‐1^+^CD8^+^T cells correlated with tumor region to a high degree, we anticipated that these TILs would reside in different clusters and that they would not achieve the same level of clonal activation as their counterparts located at other sites. Therefore, we suggest that one's ability to eradicate a tumor is not dependent on whether all CD8^+^T cells get activated in the TME, but rather the proportion of these T cells that are distributed in the optimal region of the TME.

Furthermore, we speculate that in‐depth analysis of these data, along with functional studies, will provide new insights into spatial crosstalk of the TME. Notably, tumoral BTLA^+^B cell populations enriched in the non‐central TME were associated with the distribution of PD‐1^+^CD8^+^T cells, had a close interaction and crosstalk with each other, jointly forming a special neighborhood of the TME. Conversely, the closer spatial distance of these two groups of cells was not observed in the central region. Through an in vitro assay, we showed that BTLA^+^B cells can produce IL‐6, thereby inducing PD‐1^+^CD8^+^T cells, even without antigen. In other studies, it was confirmed that the expression of BTLA was elevated in advanced tumors [23, 24, 25, 26, 27], which is consistent with our research. This phenomenon may indicate that there is a mutually synergistic mechanism between BTLA^+^CD20^+^B cells and PD‐1^+^CD8^+^T cells in the non‐central regions of the tumor, thereby further intensifying the formation of the regional immunosuppressive microenvironment.

Given that CD8^+^T cells are required for tumor eradication, we hypothesized that differences in their TCR repertoires contribute to heterogeneous antitumor immune responses. The diversity of TCR clones directly determines the recognition ability of T cells for multiple antigens, and affects their functions and the intensity of immune responses [28, 29, 30]. Studies have shown that the reduction of TCR clonal diversity is usually associated with the functional exhaustion state of T cells, especially in the TME, where T cells may tend to clonal expansion due to long‐term antigen stimulation, resulting in a decrease in diversity [31, 32, 33]. Here, analysis of TCR sequences with scRNA‐seq further provides powerful evidence that the diminished TCR repertoire variety was to a large extent restricted to the PD‐1^+^CD8^+^T subset, thereby weakening the breadth and strength of anti‐tumoral immunity initiated by anti‐PD‐1 mAb. Thus, the relationship between regional immune infiltration and TCR‐related features. Not only that, PD‐1^+^CD8^+^T cells in NSCLC are transcriptionally impaired and biased toward antigen‐driven activation, cytotoxic effector responses, and dynamic regulation of TCR and co‐receptor signaling within the TME.

With this detailed cellular heterogeneity landscape of NSCLC, our results indicated that tumor and sub‐region had substantial differences in the immune microenvironment, including cytokine, immune cell infiltration, and TCR clonality. Importantly, a combination of immune features from paired non‐central and central samples could predict patient survival more accurately compared to features from each region alone.

## Author Contributions

C.C. and Q.Q.X. conceived the idea and designed and supervised the study. W.J.J. and G.S.Y. performed the experiments and drafted the manuscript. L.L.L., F.G.Q. and S.Y. collected and analyzed data. W.J.J., G.S.Y., C.C. and Q.Q.X. analyzed data and performed statistical analysis. All authors reviewed and approved the final version of the manuscript.

## Funding

This work was supported by the National Natural Science Foundation of China (NSFC) grant 81672280 (to C.C.), the Project of Invigorating Health Care through Science, Technology and Education grant ZDXM2024002 (to C.C.), and the Project of Boxi Clinical Research grant BXLC2024004 (to C.C.).

## Ethics Statement

This study was approved by the Institutional Review Boards of The First Affiliated Hospital of Soochow University (2019‐070 and 2024‐465). The processing of clinical tissue samples is in strict compliance with the ethical standards of the Declaration of Helsinki. Informed consent was obtained from all participants.

## Conflicts of Interest

The authors declare no conflicts of interest.

## Supporting information




**Supporting File**: advs77812‐sup‐0001‐SuppMat.docx.

## Data Availability

The datasets used and/or analyzed during the current study are available from the corresponding author on reasonable request.
