# Supplementary material for: A Tumor‐Boundary Niche Enriched for PD‐1+CD8+T Cells and BTLA+B Cells Drives Resistance to Immunotherapy in NSCLC
Source: Adv Sci (Weinh). 2026 Sep 27:e77812. Online ahead of print. doi: 10.1002/advs.77812 (PMC13616405; doi:10.1002/advs.77812)
Supplement: Supplementary file 1 [file ADVS-9999-e77812-s001.docx]

**Supplemental Figure legends**

**Figure S1. Enriched GO biological process terms and Reactome pathways in genes upregulated in PD-1⁺CD8⁺T cells versus PD-1⁻CD8⁺T cells.** (A) GO BP gradient bubble plot: top 20 enriched GO BP terms ranked by gene ratio; x-axis shows gene ratio, y-axis shows terms. (B) GO BP term network graph: nodes are GO BP terms; edges reflect pairwise term similarity; node size reflects enrichment strength. (C) GO BP gene network graph: graph layout emphasizing relationships among highly overlapping terms; hub nodes represent processes with shared genes. (D) GO BP treemap: rectangles grouped by term clusters; area reflects term frequency and color reflects significance, highlighting dominant biological themes. (E) Reactome gradient bubble plot: top 20 enriched Reactome terms ranked by gene ratio; x-axis shows gene ratio, y-axis shows terms. (F) Reactome term network graph: nodes are Reactome pathways; edge reflects pairwise pathway similarity; node size reflects enrichment strength. (G) Reactome gene network graph: graph layout emphasizing relationships among highly overlapping pathways; hub nodes represent processes with shared genes. (H) Reactome treemap: clustered pathways displayed as rectangles; area reflects term frequency and color reflects significance, highlighting dominant biological themes.

**Figure S2.** Analysis of TCR-Vβ family usage in PD-1^+^ and PD-1^-^CD8^+^T cells. (A) Representative flow cytometry dot plots show each sample stained with a combination of three distinct anti-TCR-Vβ family-specific monoclonal antibodies conjugated to FITC, PE, or dual FITC/PE labels. (B) Rows represent individual participants, columns correspond to specific TCR-Vβ families, and color intensity (red scale) reflects normalized expression levels, with brighter red indicating higher values. (C) Individual usage of TCR-Vβ families in PD-1^+^ versus PD-1^−^CD8^+^T cell subsets (n = 19). Data are presented as the mean±SD. Statistical significance was determined by paired Student's t-test. *p<0.05, **p<0.01, ***p<0.001, ****p<0.0001; ns: not significant.

**Figure S3. TCR V-J usage, CDR3 length distribution, and clonal composition in PD-1⁺ and PD-1⁻CD8⁺T cells.** (A) Heatmaps showing TRA V-TRB V gene-pair frequencies in PD-1⁻CD8⁺ T cells and PD-1⁺CD8⁺T cells. (B) Heatmaps showing TRA J-TRB V gene-pair frequencies in PD-1⁻CD8⁺ T cells and PD-1⁺CD8⁺T cells. (C) Heatmaps showing TRA V-TRB J gene-pair frequencies in PD-1⁻CD8⁺T cells and PD-1⁺CD8⁺T cells. (D) Heatmaps showing TRA J-TRB J gene-pair frequencies in PD-1⁻CD8⁺T cells and PD-1⁺CD8⁺T cells. (E, F) CDR3 length distributions for combined TRA+TRB chains: histograms of amino-acid (E) and nucleotide (F) CDR3 length in PD-1⁻ (blue) versus PD-1⁺ (red) CD8⁺T cells. (G, H) Chain-specific CDR3 length distributions for TRA and TRB: histograms of amino-acid (top) and nucleotide (bottom) CDR3 length for TRA (G) and TRB (H) in PD-1⁻ (blue) versus PD-1⁺(red) CD8⁺T cells. (I) Stacked bar plot summarizing the proportional contribution of the top 5 expanded TCR clonotypes to each subset, illustrating preferential expansion of selected clones in PD-1⁺CD8⁺ T cells.

**Figure S4**. **Infiltration of PD-1^+^CD8^+^T cell subsets in NSCLC.** (A) Gating strategy for the identification of PD-1^+^CD8^+^T cells among tumor-infiltrating lymphocytes using flow cytometry. (B) Comparison of PD-1^+^CD8^+^T cell infiltration levels across patient subgroups stratified by gender, age, disease stage, and lesion size. Data are presented as mean±SD. Statistical significance was determined by Student's t-test. *p<0.05, ns: not significant.
